# Exogenous Vitamin D_3_ Modulates Response of Bovine Macrophages to *Mycobacterium avium* subsp. *paratuberculosis* Infection and Is Dependent Upon Stage of Johne’s Disease

**DOI:** 10.3389/fcimb.2021.773938

**Published:** 2022-01-17

**Authors:** Taylor L. T. Wherry, Rohana P. Dassanayake, Eduardo Casas, Shankumar Mooyottu, John P. Bannantine, Judith R. Stabel

**Affiliations:** ^1^ Infectious Bacterial Diseases, National Animal Disease Center, United States Department of Agriculture - Agricultural Research Service (USDA-ARS), Ames, IA, United States; ^2^ Department of Veterinary Pathology, Iowa State University, Ames, IA, United States; ^3^ Ruminant Diseases and Immunology, National Animal Disease Center, United States Department of Agriculture - Agricultural Research Service (USDA-ARS), Ames, IA, United States

**Keywords:** Mycobacterium avium subsp. paratuberculosis, cattle, vitamin D, macrophage, immune responses, Johne’s disease

## Abstract

*Mycobacterium avium* subspecies *paratuberculosis* (MAP), the causative agent of ruminant enteritis, targets intestinal macrophages. During infection, macrophages contribute to mucosal inflammation and development of granulomas in the small intestine which worsens as disease progression occurs. Vitamin D_3_ is an immunomodulatory steroid hormone with beneficial roles in host-pathogen interactions. Few studies have investigated immunologic roles of 25-hydroxyvitamin D_3_ (25(OH)D_3_) and 1,25-dihydroxyvitamin D_3_ (1,25(OH)_2_D_3_) in cattle, particularly cattle infected with MAP. This study examined the effects of exogenous vitamin D_3_ on immune responses of monocyte derived macrophages (MDMs) isolated from dairy cattle naturally infected with MAP. MDMs were pre-treated with ± 100 ng/ml 25(OH)D_3_ or ± 4 ng/ml 1,25(OH)_2_D_3_, then incubated 24 hrs with live MAP in the presence of their respective pre-treatment concentrations. Following treatment with either vitamin D_3_ analog, phagocytosis of MAP by MDMs was significantly greater in clinically infected animals, with a greater amount of live and dead bacteria. Clinical cows had significantly less CD40 surface expression on MDMs compared to subclinical cows and noninfected controls. 1,25(OH)_2_D_3_ also significantly increased nitrite production in MAP infected cows. 1,25(OH)_2_D_3_ treatment played a key role in upregulating secretion of pro-inflammatory cytokines IL-1β and IL-12 while downregulating IL-10, IL-6, and IFN-γ. 1,25(OH)_2_D_3_ also negatively regulated transcripts of *CYP24A1*, *CYP27B1*, *DEFB7*, *NOS2*, and *IL10*. Results from this study demonstrate that vitamin D_3_ compounds, but mainly 1,25(OH)_2_D_3_, modulate both pro- and anti-inflammatory immune responses in dairy cattle infected with MAP, impacting the bacterial viability within the macrophage.

## 1 Introduction

Johne’s disease in ruminants, caused by the intracellular pathogen *Mycobacterium avium* subsp. *paratuberculosis* (MAP), is characterized by a chronic enteritis leading to clinical symptoms of watery diarrhea and wasting due to malabsorption of nutrients by a thickened intestinal wall (Manning and Collins, 2001). Economic impacts vary depending on the prevalence of MAP-positive animals in the herd, but losses can reach upwards of $245/cow annually (Ott et al., 1999). To mitigate losses due to high culling rates and decreased milk yield, it is important to understand the highly specialized mechanisms of immune system evasion and manipulation MAP utilizes that allows for propagation of disease.

Macrophages are key reservoirs for MAP and provide a niche microenvironment in which the pathogen can replicate while concealing itself from innate and adaptive immune responses within the infected host. Initial recognition of mycobacteria species by APCs has been suggested to be mediated by mycobacterial DNA binding TLR9 (Bafica et al., 2005; Arsenault et al., 2013), and bacterial cell wall lipoproteins interacting with TLR2 (Weiss et al., 2008), which forms heterodimers with TLR1 or TLR6 (Jin et al., 2007; Kang et al., 2009). Additionally, mutations in TLR1, TLR2, and TLR4 have been found to be associated with increased susceptibility to MAP infection in cattle, possibly as a result in dampened pro-inflammatory cytokine responses such as IFN-γ and IL-12 (Bhide et al., 2009; Mucha et al., 2009). To successfully establish chronic infection, MAP employs a variety of tools. Studies have shown MAP’s ability to block signaling of IFN-γ and TNF-α, which are both cell-activating pro-inflammatory cytokines that play a role in inhibiting intracellular replication of MAP (Stabel, 1995; Arsenault et al., 2012). MAP may control pro- and anti-apoptotic events within monocytes and macrophages, which perhaps functions to regulate replication and dissemination of the bacteria since apoptosis and engulfment by clean-up macrophages would perpetuate the infection cycle (Allen et al., 2001; Kabara and Coussens, 2012; Periasamy et al., 2013). Most importantly, MAP can arrest phagosome-lysosome fusion, thereby preventing the acidification of the compartment and subsequent pathogen destruction (Sturgill-Koszycki et al., 1994; Hostetter et al., 2003; Weiss et al., 2004). Thus, a delicate balance of both pro-inflammatory and regulatory cytokine action is needed to prevent progression to advanced disease states by maintaining control of the infection while regulating inflammation in the host tissue.

The dynamics of macrophage activation and polarization to a host defense (M1) or resolution/repair (M2) phenotype plays a significant role in the progression of bovine paratuberculosis. Classically activated M1 macrophages possess a pro-inflammatory cytokine repertoire, commonly defined by production of IFN-γ, TNF-α, IL-12, IL-1β, and nitric oxide (Benoit et al., 2008). In contrast, alternatively activated M2 macrophages can be identified through the production of anti-inflammatory mediators such as IL-4, IL-10, and IL-13. In intestinal tissue from MAP infected cattle, subclinical infections have a similar ratio of M1 to M2 macrophage phenotypes, likely contributing to control of the infection, while clinically infected animals skewed towards an M2 phenotype (Jenvey et al., 2019). This imbalance between classically activated M1 and alternatively activated M2 macrophages in clinical animals is often accompanied by a failure of pro-inflammatory T helper 1 (Th1) responses in later stage clinical disease, contributing to an increased bacterial load and intestinal pathology (Khalifeh and Stabel, 2004; Khare et al., 2016). Both cell mediated and humoral responses to pathogen infection are dependent upon the co-stimulatory signal resulting from T cell CD40 ligand (CD40L, CD154) ligation with CD40 on antigen presenting cells (Yamauchi et al., 2000; Elgueta et al., 2009).

The non-classical immunomodulatory effects of vitamin D_3_ on host-pathogen interactions have been well established in human and veterinary medicine (Crowle et al., 1987; Waters et al., 2001; Lippolis et al., 2011). Cells of the immune system have the capability to convert circulating 25-dihydroxyvitamin D_3_ (25(OH)D_3_) to its bioactive form, 1α-dihydroxyvitamin D_3_ (1,25(OH)_2_D_3_) (Adams et al., 1983; Nelson et al., 2010b), which in turn modulates immune system signaling during infection. This local conversion is achieved through the action of 1α-hydroxylase, also known as CYP27B1 (García-Barragán et al., 2018). The precursor 25(OH)D_3_ is found in the circulation at a concentration 1,000 times greater than 1,25(OH)_2_D_3_ and possesses a longer biological half-life of 15 days compared to 4-6 hrs, respectively (Horst et al., 1981; Jones, 2008). Evidence of local regulation of vitamin D_3_ conversion within bovine monocytes and macrophages has been established *in vitro* (Nelson et al., 2010b) and *in vivo* (Nelson et al., 2010a). Additionally, foundational work in human tuberculosis, caused by *Mycobacterium tuberculosis* (*M. tb*), revealed the requirement of 1,25(OH)_2_D_3_ action to upregulate antimicrobial peptide cathelicidin following TLR2/1 heterodimer signaling in human monocytes and macrophages (Liu et al., 2006; Liu et al., 2007). Activation of cathelicidin by 1,25(OH)_2_D_3_ resulted in decreased intracellular viability of *M. tb* through upregulation of vitamin D receptor (VDR) and CYP27B1. Species differences exist, with humans possessing a single cathelicidin gene compared to 11 cathelicidin genes in cattle, upon which no effects of 1,25(OH)_2_D_3_ have thus far been observed (Nelson et al., 2010b).

Outside of cathelicidin responses in cattle, vitamin D_3_ influences other inflammatory responses following antigenic exposure. Stimulation of bovine monocytes with lipopolysaccharide (LPS) and treatment with 1,25(OH)_2_D_3_ has been shown to enhance pro-inflammatory gene expression responses, such as IL-1β (*IL1B)* and inducible nitric oxide synthase (iNOS/*NOS2*) (Nelson et al., 2010b). A corresponding increase in nitrite concentration was also observed, along with upregulated gene expression for the chemokine RANTES/*CCL5* (regulated upon activation, normal T cell expressed and secreted) (Nelson et al., 2010b). Furthermore, evidence of vitamin D_3_ exhibiting immunomodulatory roles in cattle during exposure to mycobacterial species has been presented in recent years. Monocyte derived macrophages infected with *Mycobacterium bovis* (*M. bovis*) and treated with 1,25(OH)_2_D_3_ enhanced nitric oxide production and its corresponding *NOS2* gene expression (García-Barragán et al., 2018). Additionally, peripheral blood mononuclear cells (PBMCs) from calves vaccinated with *M. bovis*-BCG and treated with 1,25(OH)_2_D_3_ or 25(OH)D_3_ observed increased *NOS2* and nitric oxide, while concurrently decreasing IFN-γ responses following re-exposure to *M. bovis* purified protein derivative (PPD) (Nelson et al., 2011).

The objective of this study was to characterize the effects of vitamin D_3_ on immune responses within cattle at different stages of MAP infection while incorporating T cell and macrophage interactions *in vitro*. To the best of our knowledge, this is the first study to report comprehensive immune responses to MAP accompanied by vitamin D_3_ treatment at specific stages of Johne’s disease. Based on previous evidence of immunomodulation induced by vitamin D_3_, especially in cases of bacterial infection, we hypothesized in the present study that 25(OH)D_3_ or 1,25(OH)_2_D_3_ treatment of bovine MDM-PBMC co-cultures would elicit protective immune responses that contribute to disease resolution upon re-exposure to MAP.

## 2 Materials And Methods

### 2.1 Animals

Holstein dairy cows used in this study were housed separately on-site according to positive or negative infection status with MAP to prevent cross-contamination. All experimental procedures were approved by the IACUC (National Animal Disease Center, Ames, IA). Housing facilities are accredited by the American Association for Accreditation of Laboratory Animal Care.

Age ranges for each set of experiments are as follows: *Bac*Light viability (2 - 11 yrs); activation markers (2.5 – 11.5 yrs); cytokine induction (3 – 14 yrs). Diagnostic tests measuring serum MAP-specific antibody levels (Herdchek; IDEXX, Westbrook, ME), bovine IFN-γ plasma levels (Bovigam; Prionics, La Vista, NE), and fecal shedding detected by culture on Herrold’s egg yolk medium (Becton Dickinson, Sparks, MD) were used to categorize cows into stage of MAP infection, as previously described (Stabel and Bannantine, 2019).

For the *Bac*Light viability assay experiments, clinical cows (n=8) were culture positive for MAP shedding in the feces. This group was ELISA positive for MAP serum antibody having an average S/P ratio of 1.36 along with an average MAP-specific IFN-γ recall response of OD_450_ 0.43 ± 0.22 (Abs_450nm_MPS-Abs_450nm_NS). Subclinical cows (n=8) were ELISA negative for MAP serum antibodies and IFN-γ OD_450_ results averaged 0.15 ± 0.05. Control animals (n=8) were negative for all MAP diagnostic tests.

Clinical cows (n=7) in the macrophage activation marker experiment were ELISA positive for MAP serum antibody having an average S/P ratio of 1.9, and had a MAP-specific IFN-γ recall response of OD_450_ 0.90 ± 0.31 (Abs_450nm_MPS-Abs_450nm_NS). All cows but one in this group were culture positive for MAP fecal shedding, with an average of 31 CFU/g fecal matter. Subclinical cows (n=8) were ELISA negative for MAP serum antibodies and IFN-γ OD_450_ results averaged 0.84 ± 0.30. Two cows in this group were culture positive for MAP shedding in the feces, but shedding was low at < 9 CFU/g fecal matter. The control group (n=6) was negative for all MAP diagnostic tests.

Lastly, diagnostic values for the cytokine induction experiments included clinical cows (n=7) that were ELISA positive for MAP serum antibody, with an average S/P ratio of 1.27. The average MAP-specific IFN-γ recall response for this group was OD_450_ 0.39 ± 0.17 (Abs_450nm_MPS-Abs_450nm_NS). Clinical cows were culture positive for MAP having an average of 189 CFU/g fecal matter. The subclinical group (n=7) was ELISA negative for MAP serum antibodies and had an average IFN-γ OD_450_ of 0.26 ± 0.11. Three subclinical cows were fecal culture positive for MAP and had an average shedding value of 12 CFU/g fecal matter. Control cows (n=9) were negative for all MAP diagnostic tests.

### 2.2 MAP 167 Culture

This study utilized a virulent strain of MAP (clinical cow 167; NADC, Ames, IA) isolated from a dairy cow in the clinical stage of Johne’s disease. MAP 167 cultures were prepared by inoculating a frozen aliquot into 450 ml of sterile Middlebrook 7H9 broth (Becton Dickinson, Franklin Lakes, NJ) at pH 5.9, supplemented with 1 mg mycobactin J (Allied Monitor Inc., Fayette, MO), 50 ml oleic acid-albumin-dextrose complex (OADC; Becton Dickinson), and 0.05% Tween 80 (Sigma, St. Louis, MO). Bacterial cultures were incubated at 39°C until they reached the logarithmic growth phase at an optical density of 0.2 to 0.4 at 540 nm (OD_540_). Aliquots were prepared and stored in D-PBS (Sigma-Aldrich) as described previously (Bradner et al., 2013).

### 2.3 Vitamin D_3_ Stock Preparation

25(OH)D_3_ and 1,25(OH)_2_D_3_ stocks were supplied in pure ethanol by Dr. T. A. Reinhardt (NADC, Ames, IA). Working stocks of 25(OH)D_3_ and 1,25(OH)_2_D_3_ were made by diluting in 100% fetal bovine serum to a concentration of 1000 ng/ml and 40 ng/ml, respectively. Following a final 1:10 dilution, cell culture treatment wells had a final concentration of 10% FBS with either 100 ng/ml 25(OH)D_3_ or 4 ng/ml 1,25(OH)_2_D_3_. Final ethanol concentrations for 25(OH)D_3_ and 1,25(OH)_2_D_3_ treatments did not exceed 0.11% or 0.05%, respectively. All stocks were stored in airtight glass vials at -20°C and kept protected from light during storage and experimental procedures.

### 2.4 PBMC Isolation and MDM Culture

PBMCs were isolated from whole blood drawn from jugular venipuncture into 2× acid-citrate-dextrose (in-house, 1:10). Whole blood diluted 1:2 in D-PBS (Sigma-Aldrich, St. Louis, MO) was centrifuged 800 × g for 30 min and the resulting buffy coat fraction was laid over Histopaque -1077 (Sigma) for density centrifugation. PBMCs underwent lysing-restoring steps to remove red blood cell contamination, washed in D-PBS, and resuspended in complete growth medium (cRPMI; RPMI-1640 with L-glutamine and HEPES [Gibco, Grand Island, NY], 1% antibiotic-antimycotic [100 U/ml penicillin, 100 μg/ml streptomycin, 250 ng/ml Amphotericin B, Gibco], 1% MEM non-essential amino acids solution [100×, Gibco], 2% MEM essential amino acids solution [50×, Gibco], 2 mM L-glutamine [200 mM, Gibco]; 1% sodium pyruvate [100mM, Gibco]; and 50 μM 2-mercaptoethanol [50 mM, Gibco]) supplemented with 10% (v/v) heat inactivated fetal bovine serum (FBS, HyClone Cytiva, Marlborough, MA). Live cells were counted using trypan blue dye exclusion and a TC20 automated cell counter (Bio-Rad, Hercules, CA). Cell densities were adjusted to 4.0 × 10^6^ cells per ml in cRPMI with 10% FBS and seeded at 1 ml onto 24 well ibi-treat μ-plates (Ibidi, Fitchburg, WI) for activation marker analysis, 4 well ibi-treat μ-slides (Ibidi) for *Bac*Light viability staining and propidium monoazide dye (PMAxx) viability analysis, or 24 well flat-bottom plates (Becton Dickinson, Franklin Lakes, NJ) for cytokine secretion and expression assays. PBMCs were incubated 5-6 days in a 39°C humidified incubator to generate MDMs.

### 2.5 Vitamin D_3_ Treatment and MAP Inoculation

Cell cultures had their supernatants replaced on day 5 to pre-treat with 1 ml cRPMI containing 10% FBS and 100 ng/ml 25(OH)D_3_ for 24 hrs. Untreated control replicates also had cell culture medium replaced on this day with 1 ml cRPMI containing 10% FBS only. On day 6, another set of replicate wells were pre-treated with 4 ng/ml 1,25(OH)_2_D_3_ for 6 hrs. During this incubation, separate culture plates designated for counting MDMs were gently washed twice with 1 ml room temperature D-PBS, then incubated on ice for 15 min after addition of 1 ml cold D-PBS to promote non-mechanical detachment of adherent cells. MDMs were then removed from culture plates and counted using trypan blue dye exclusion on a TC20 automated cell counter. Completion of vitamin D_3_ pre-treatments were timed to intersect on day 6. At this time, all wells had fresh media replaced with their respective treatments, and those assigned for MAP infection were inoculated at a 10:1 multiplicity of infection (MOI) ± 25(OH)D_3_ or 1,25(OH)_2_D_3_ in 1 ml cRPMI with 10% FBS. A 10:1 MOI has previously been reported as an ideal ratio for optimal uptake of MAP by macrophages and increasing MOI does not show a significant benefit (Murphy et al., 2006; Chiang et al., 2007). All plates were then incubated 24 hrs at 39°C.

Plates assigned for capture of cell culture supernatants and RNA extraction underwent the same vitamin D_3_ treatments and MAP inoculation protocol as previously described, but prior to addition of *in vitro* treatments, plates were centrifuged 1500 RPM (491 × g) for 5 min to retain lymphocytes to allow for macrophage cross-talk with T cells.

For the MAP viability assays PBMCs were harvested from cows at two time points, therefore, separate experiments were performed for 1,25(OH)_2_D_3_ and 25(OH)D_3_ treatments. Cytokine induction and macrophage activation marker analysis were also two separate experiments but had 1,25(OH)_2_D_3_ and 25(OH)D_3_ treatments administered concurrently. Vitamin D_3_ treatment concentrations used in these experiments were selected based on previous work in cattle (Nelson et al., 2010b; Nelson et al., 2011).

### 2.6 Activation Marker Fluorescence Microscopy

MDMs cultured in coverslip-bottom 24 well plates (Ibidi) were first blocked in serum-free block buffer (X0909, Agilent Technologies, Santa Clara, CA) for 30 min at room temperature. Extracellular targets were all incubated 1 hr at room temperature in the dark, beginning with CD40 (BOV2107; Washington State University, Pullman, WA), goat anti-mouse biotin labeled secondary IgG (31802; Invitrogen), and fluorescently labeled tertiary NeutrAvidin DyLight 650 (84607, Invitrogen). MHCII primary (BOV2004; Washington State University) and goat anti-mouse IgG2a AF488 secondary antibodies (A21131; Invitrogen) were added, followed by cell fixation for 15 min with paraformaldehyde diluted to 1% (157-4; Electron Microscopy Sciences, Hatfield, PA). Cells were washed and permeabilized with 20 mM MOPS with 1mM MgCl2, 150 mM NaCl, and 0.1% Saponin (perm buffer) three times for 5 min each in preparation for labeling intracellular targets. Cells were blocked again for 30 min, incubated with pan macrophage marker CD68 primary antibody (M0718; Agilent Technologies), then AF594 goat anti-mouse IgG1 (115-585-205; Jackson Labs, West Grove, PA) for 1 hr each. Counter-staining was achieved with 1 μg/ml DAPI and approximately 250 μl of non-hardening mounting medium (50001; Ibidi) was added to each well for imaging.

Images were acquired with a 40× Nikon Plan Fluor N.A. 1.3 objective using oil immersion with 6.2 second pixel dwell time on a Nikon A1 Resonance Plus confocal microscope using NIS-Elements Advanced Research software v5.11 (Nikon, Melville, NY). The instrument contains a 4-laser gallium-arsenide-phosphide/normal photomultiplier tube (GaAsP PMT) fluorescence detector unit (A1-DU4) with two GaAsP PMTs (488 and 561 nm) and two normal PMTs (405 and 640). Fluorescent signal was detected sequentially using the following solid-state diode lasers and bandpass filters: 405 nm (450/50 nm), 488 nm (525/50 nm), 561 nm (600/50 nm), and 640 nm (685/70 nm). A minimum of 10 images were acquired per cow and treatment. Following image acquisition, binary layers for each laser channel were created with thresholds established from no stain and secondary only controls to exclude any nonspecific background. The mean fluorescence intensity (MFI) of CD40 and MHCII fluorescence signal was measured on MDMs, identified as expressing CD68. Analysis was run on unaltered images. The representative image chosen for presentation had lookup tables applied within the Nikon NIS-Elements software to brighten signal and was post-processed in Adobe Photoshop (version 22.0; San Jose, CA) to further increase brightness and reduce shadows for better print viewing. All alterations were uniformly applied to the entire image.

### 2.7 MAP Viability Assessment

#### 2.7.1 BacLight

MDMs were washed with 20 mM MOPS containing 1mM MgCl_2_ and 150 mM NaCl (wash buffer). To label any remaining extracellular MAP for analysis exclusion, an anti-MAP rabbit polyclonal (#272, in-house) antibody was added, followed by an Alexa Fluor 647 (AF647) goat-anti rabbit secondary antibody (A21244; Invitrogen, Carlsbad, CA). SYTO 9 and propidium iodide (P.I.) dyes supplied in the *Bac*Light viability kit (L7012; Life Technologies, Carlsbad, CA) were each diluted according to the manufacturer’s recommendations in perm buffer. Saponin detergent in this buffer facilitated intracellular staining of MAP through entry of P.I. into the live bovine MDMs, which can only enter cells in the event membrane integrity is compromised. MDMs were incubated in 0.5 ml of *Bac*Light dye solution for 15 min at room temperature in the dark. The viability dye was removed, and 1 ml of wash buffer was added for live cell confocal imaging.

Similar to the activation marker fluorescence assay, a minimum of 10 images were taken per cow and treatment. Due to the permeabilization of live bovine MDM cell membranes previously discussed, MDM nuclei were fluorescently labeled along with MAP genomic material. P.I. exhibits a stronger binding affinity for nucleic acids than SYTO 9 (Stocks, 2004) and therefore labeled all mammalian nuclei in each sample. When binary layers were created for each laser channel during analysis with the Nikon NIS-Elements software, thresholds were established from no stain and secondary antibody only controls to exclude background fluorescence, and size limits were applied in the 488 nm and 594 nm channel to treat equally the channels measuring intracellular MAP. The utility of setting size parameters was to exclude large mammalian nuclei labeled with P.I. and allowed for quantification of MAP only. Additionally, MAP co-labeling with P.I. or SYTO 9 and AF647 secondary antibody signal were identified as extracellular and thus excluded from the intracellular MAP viability calculation (area μm^2^). Live and dead MAP area were calculated by the software measuring binary area pixels expressing fluorescence in the 488 nm and 594 nm channels, respectively. Lookup tables were applied to the representative image selected to brighten signal and was post-processed in Adobe Photoshop (version 22.0; San Jose, CA) to further increase brightness and reduce shadows for better print viewing. All alterations were uniformly applied to the entire image.

#### 2.7.2 Propidium Monoazide Dye (PMAxx)

After incubation with MAP, MDMs were washed with room temperature D-PBS (Sigma-Aldrich) then permeabilized with 0.1% saponin in DNase-free dH_2_O (Gibco) for 30 min at 37°C in a humidified incubator. MDMs were removed by vigorously pipetting and transferred to a microcentrifuge tube. Samples were pelleted by centrifugation at 4,500 RPM for 30 min, then the supernatant was removed and cell pellet was resuspended in 1 ml DNase-free dH_2_O. Samples were centrifuged again at 20,000 × g for 10 min, supernatant removed, and cell pellet resuspended in 500 μl dH_2_O. Propidium monoazide (PMAxx) dye was added to each sample at a final concentration of 25 μM, vortexed to ensure homogeneity of the solution, and incubated 10 min in the dark at room temperature on a rocker. Cross-linking of the PMAxx dye and free nonviable MAP DNA was achieved by exposing samples to LED light for 15 min using a PMA-Lite Photolysis Unit (Biotium, Fremont, CA). Samples were then centrifuged 16,000 × g for 10 min and the supernatant was discarded. Samples were stored at -20°C until ready for further processing.

Total DNA was purified from the crude cell lysates using the Qiagen DNeasy Blood & Tissue kits according to the manufacturer’s protocol. DNA was eluted in a final volume of 100 μl and stored at -20°C until ready for qPCR. MAP-specific IS900 primers and probes were used to measure the amount of viable MAP within the harvested MDMs and their sequences are as follows: forward 5′-CCGCTAATTGAGAGATGCGATTGG-3′; reverse 5′-AATCAACTCCAGCAGCGCGGCCTCG-3′; and probe 5′-FAM-TCCACGCCCGCCCAGACAGG-TAMRA-3′. Real-time qPCR was performed on a sample volume of 25 μl using TaqMan Environmental Master Mix 2.0 and a thermal profile of 1 cycle for 2 min at 50°C, 1 cycle for 10 min at 95°C, followed by 40 cycles of denaturation at 94°C for 25 sec and 1 min at 60°C for primer annealing and extension. Each sample was plated in triplicate and viable CFUs were calculated from a standard curve ranging from 1ng/μl to 1 fg/μl stock MAP DNA.

### 2.8 RNA Extraction and cDNA Synthesis

On day 7, cell culture plates were centrifuged for 10 min at 500 × g. Cell supernatants were collected and stored in 1.5 ml microcentrifuge tubes (Axygen, Union City, CA) at -80°C for later cytokine secretion analysis. The cells remaining in each well were transferred to separate 1.5 ml microcentrifuge tubes (Axygen) and stored in 350 μl RNAprotect Cell Reagent (Qiagen, Hilden, Germany) at -80°C for further processing. Beginning RNA extraction, samples were thawed and centrifuged 5,000 x g for 5 min. The cell pellet was lysed in 600 μl Buffer RLT Plus (Qiagen) supplied in the RNeasy Mini kit. RNA purification was performed on columns supplied in the kit according to the manufacturer’s instructions. RNA elution was achieved using a final volume of 60 μl RNase-free water also supplied in the kit. The RNA 6000 Nano kit (Agilent, Santa Clara, CA) and 2100 Bioanalyzer instrument (Agilent) were used to quantify total RNA present. Samples were diluted to 12.5 ng/μl in 40 μl RNAse-free water and if the RNA concentration fell below 12.5 ng/μl, samples were concentrated using a SpeedVac DNA 120 and re-analyzed. Superscript IV (Invitrogen, Carlsbad, CA) was used to reverse transcribe RNA. The reaction mixture included a final concentration of ~175 ng random hexamer primers (Invitrogen), ~600 nM of each dNTP (Invitrogen), and 2,000 units of Superscript IV. Primers were annealed for 5 min at 65°C, followed by incubation with the reverse transcriptase enzyme for 10 min at 23°C, 10 min at 50°C, then 10 min at 80°C per the manufacturer instructions. Stock cDNA was diluted 1:10 in RNase and DNase-free water (Gibco) and stored at -20°C.

### 2.9 Cytokine Gene Expression Real-Time qPCR

TaqMan bovine gene expression assays (Applied Biosystems, Foster City, CA) listed in **Table 1** were used to quantify relative expression of IL-1β (*IL1B*), IL-10 (*IL10*), IL-12A (*IL12A*), IL-17A (*IL17A*), β-defensin 7 (*DEFB7*), β-defensin 10 (*DEFB10*), CYP24A1 (*CYP24A1*), CYP27B1 (*CYP27B1*), IFN-γ (*IFNG*), iNOS (*NOS2*), RANTES (*CCL5*), and TNF-α (*TNF*) in cells from the 24 hr cytokine supernatant samples. The following protocol was optimized previously in our lab^1^. Samples were plated in duplicate with a reaction mixture consisting of 10 μl TaqMan Fast Advanced Master Mix (Applied Biosystems), 1 μl gene expression assay, 5 μl nuclease-free water, and 4 μl cDNA template per well. Relative quantitation (RQ) values were calculated by normalization to 18S rRNA expression (FAM/MGB probe, non-primer limited; Applied Biosystems) and calibration to the NS sample. Data were analyzed using the 2^−ΔΔCT^ method (Livak and Schmittgen, 2001).

**Table 1:** ThermoFisher Scientific gene expression assays.

Target	Gene Alias	Assay ID	Target Sequence
IL-1β	*IL1B*	Bt03212742_m1	ACAGATGAAGAGCTGCATCCAACAC
IL-10	*IL10*	Bt03212725_g1	CTGGATGACTTTAAGGGTTACCTGG
IL-12A	*IL12A*	Bt03213918_m1	GCTACAGAAGGCCAGACAAACTCTA
IL-17A	*IL17A*	Bt03210251_m1	ACTTCATCTATGTCACTGCTACTGC
β-defensin 7	*DEFB7*	Bt04318496_mH	TGTCTGCTGGGTCAGGATTTACTCA
β-defensin 10	*DEFB10*	Bt03415224_m1	TGTCTTCTGGGTCAGGATTTACTCA
24-hydroxylase	*CYP24A1*	Bt04306549_g1	AAAGGAATTGTCCGCAAATACGACG
1α-hydroxylase	*CYP27B1*	Bt04311111_g1	GGATTGCTCACCGCGGAAGGGGAAG
IFN-γ	*IFNG*	Bt03212722_g1	ATTGGAAAGATGAAAGTGACAAAAA
iNOS	*NOS2*	Bt03249590_m1	CAGCCCCCGTCCAGTCCAGTGACAC
RANTES	*CCL5*	Bt03216832_m1	CTCCATGGCAGCAGTTGTCTTTATC
TNF-α	*TNF*	Bt03259155_g1	CAAACACTCAGGTCCTCTTCTCAAG

### 2.10 Cytokine Secretion

A custom Milliplex bovine 8-plex cytokine/chemokine magnetic bead panel was used to quantify cytokine concentrations from MDM culture supernatants and included IFN-γ, IL-1β, IL-6, IL-10, IL-17A, IL-36RA, MCP-1, and TNF-α. Samples were loaded in duplicate onto 96-well plates and incubated with beads overnight (16-18 hrs) at 4°C on a plate shaker protected from light. Biotinylated detection antibodies were added to each sample, followed by Streptavidin-Phycoerythrin. A magnetic plate was used to retain beads during manual washing. Drive Fluid was used to resuspend beads in preparation for running samples on the Luminex MAGPIX xMAP instrument. Individual cytokines were identified through fluorescent signal emitted by internal bead dye ratios pre-determined by the manufacturer. Phycoerythrin fluorescence was used to generate cytokine concentrations present in each sample by comparison to a standard curve. Data were collected and summarized using the Bio-Plex Manager software (Bio-Rad).

IL-12 concentrations were quantified by ELISA standard curve generated using a bovine IL-12/IL-23 p40 recombinant protein (RP0077B; Kingfisher). Capture antibody (MCA1782EL, Bio-Rad) and biotinylated detection antibody (MCA2173B, Bio-Rad) were used at concentrations of 1.1 μg/ml and 0.5 μg/ml, respectively. Colorimetric changes were developed through the addition of HRP bound streptavidin, followed by incubation with TMB substrate. Signal was detected at 450nm using a SpectraMax 340PC384 microplate reader (Molecular Devices, San Jose, CA). The Milliplex and IL-12 standard ELISA protocols were optimized previously in our lab (Wherry et al., 2021).

### 2.11 Nitrite Quantification

The Griess reagent system (Promega, Madison, WI) was used to measure nitrite production in supernatants from day 7 MDM cultures following 24 hr incubation with MAP. The reaction was performed by preparing a standard curve from 100 μM to 1.56 μM of nitrite, then incubating 50 μl sulfanilamide solution with 50 μl of experimental samples for 10 min at room temperature in the dark. Lastly, 50 μl of *N*-(1-napthyl)ethylenediamine dihydrochloride (NED) solution was added. After incubating for 10 min in the dark, absorbance was measured at 540nm using a SpectraMax 340PC384 microplate reader (Molecular Devices).

### 2.12 Statistical Analysis

The *Bac*Light dataset was analyzed using the procedure MIXED from SAS version 9.4 (SAS Institute, Cary, NC). For all other assays, statistical analysis was performed using R Statistical Software (version 4.0.3, R Foundation for Statistical Computing, Vienna, Austria) and RStudio (version 1.3.1093, Boston, MA). Statistical models were built using the mixed model function “lme” from package “nlme” (Pinheiro et al., 2021) and *post-hoc* tests were performed using the package “emmeans” (Lenth, 2021) with a Tukey adjustment for multiple comparisons. Relative gene expression analysis was performed using ΔΔCt values and data were transformed using the 2^-ΔΔCt^ method for graphical representation.

## 3 Results

### 3.1 Intracellular MAP Viability

**Figure 1:**
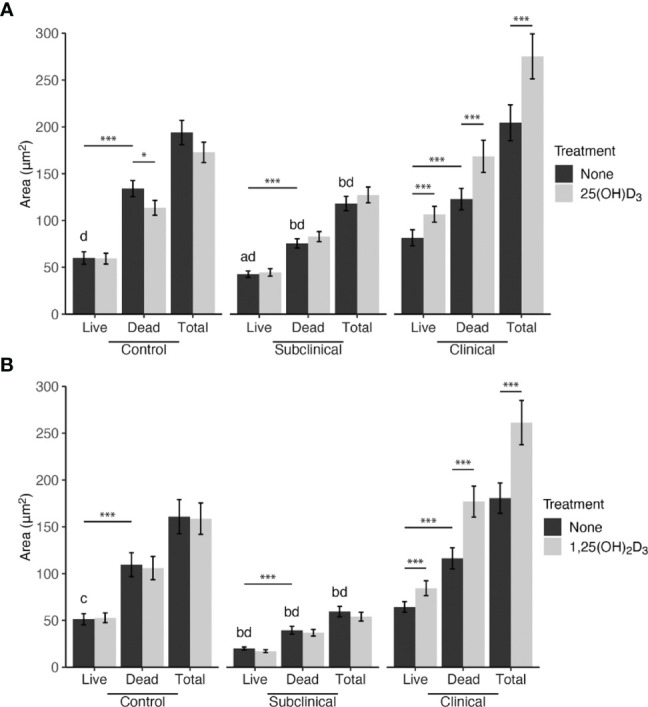
Mean fluorescence area of *Mycobacterium avium* subsp. *paratuberculosis* (MAP) present within monocyte-derived macrophages (MDMs) cultured from naturally infected dairy cattle (subclinical n=8, clinical n=8, and noninfected controls n=8). Peripheral blood mononuclear cells (PBMCs) were cultured 5-6 days to generate MDMs, pre-treated with vitamin D_3_ as detailed in methods, then incubated 24 hrs with live MAP at 10:1 MOI **(A)** ± 25(OH)D_3_ or **(B)** ± 1,25(OH)_2_D_3_. Live and dead MAP were determined using SYTO 9 and propidium iodide, respectively. Data are presented as the mean ± SE. Intra-status comparison significance levels are *< 0.05, **< 0.01, ***< 0.001. Intra-viability comparisons against the control group are a < 0.01, b < 0.001 and intra-viability comparisons against the clinical group are c < 0.05 and d < 0.001.

**Figure 2:**
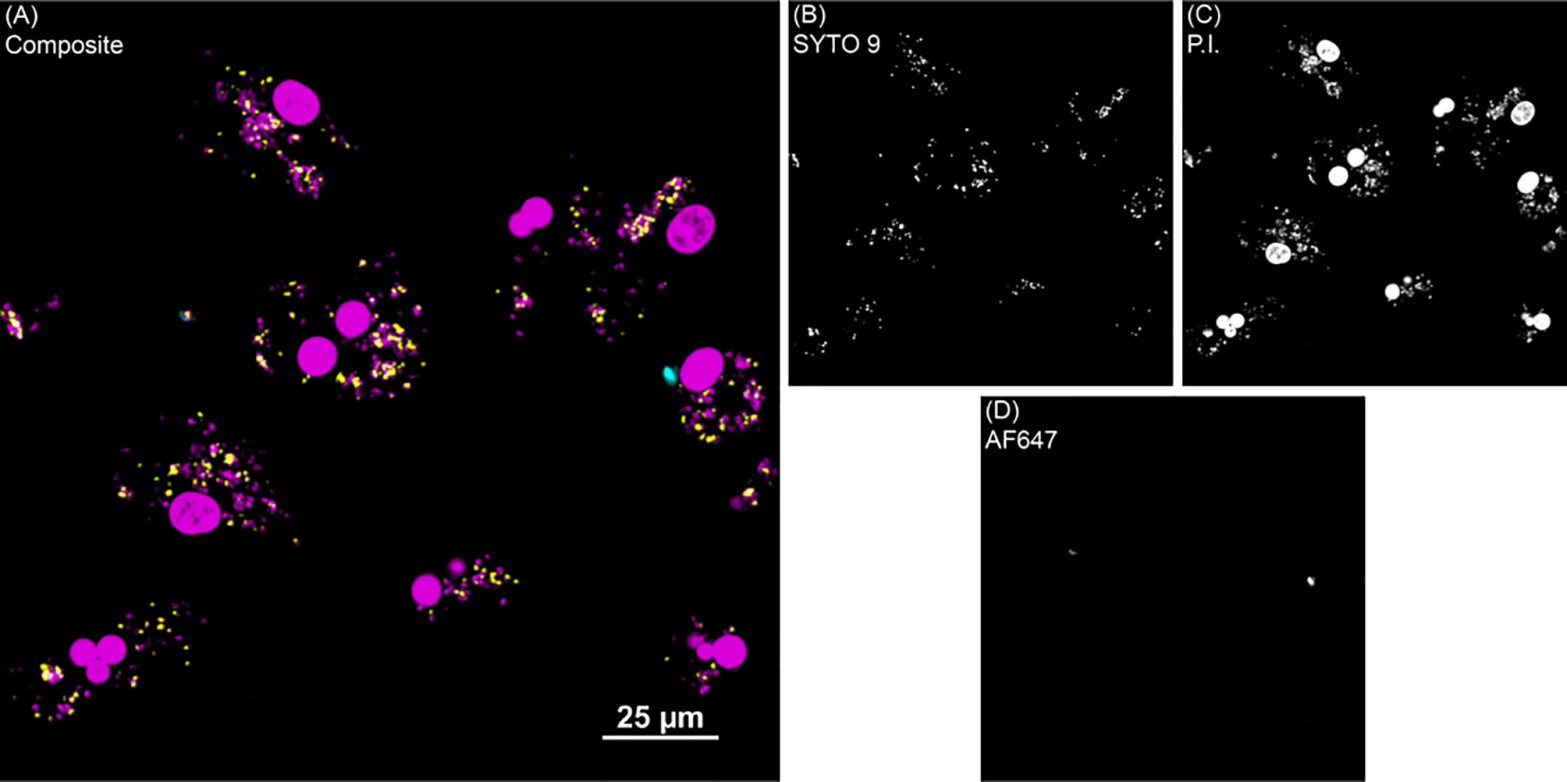
*Bac*Light fluorescence to investigate intracellular MAP viability. **(A)** Shows one representative image of monocyte derived macrophage culture without vitamin D_3_ treatment from a clinical cow. **(B)** SYTO 9 (yellow) represents viable intracellular MAP detected in the 488 nm channel. **(C)** Propidium iodide (P.I.) was used to label intracellular nonviable MAP in the 561 nm channel (magenta). Large, dense magenta spheres are nuclei and were excluded from analysis based on size. **(D)** Extracellular MAP (cyan) that was not washed away was detected in the 640 nm channel, and its dual labeling with P.I. or SYTO 9 flagged those bacteria for exclusion from analysis.

The effects of exogenous vitamin D_3_ compounds on killing of MAP were assessed in MDM cultures by *Bac*Light viability dyes following incubation with MAP (10:1 MOI) for 24 hrs. Overall, treatment with both 25(OH)D_3_ and 1,25(OH)_2_D_3_ resulted in significant (*P* < 0.001) increases in live, dead, and total MAP in MDMs from clinical cows (**Figure 1**). In addition, significantly (*P* < 0.001) higher numbers of dead MAP were found within untreated MDMs from each infection group. The amount of total MAP phagocytized by MDMs was observed to be significantly (*P* < 0.001) greater in clinical animals, while subclinical cows demonstrated a decrease in MAP uptake compared to noninfected controls (*P* < 0.001). There was no significant impact of vitamin D_3_ treatment on viability and total MAP in control or subclinical MDMs, except for a reduction (*P* < 0.05) in dead MAP for the control group that underwent 25(OH)D_3_ treatment (**Figure 1A**). Lastly, a higher number of live MAP were present in untreated MDMs from clinical animals compared to control animals for both the 25(OH)D_3_ (*P* < 0.001) and 1,25(OH)_2_D_3_ (*P* < 0.05; **Figure 1B**) experiments, an effect that contributed to the overall increase in total intracellular MAP. A representative *Bac*Light fluorescence image is shown in **Figure 2**.

**Figure 3:**
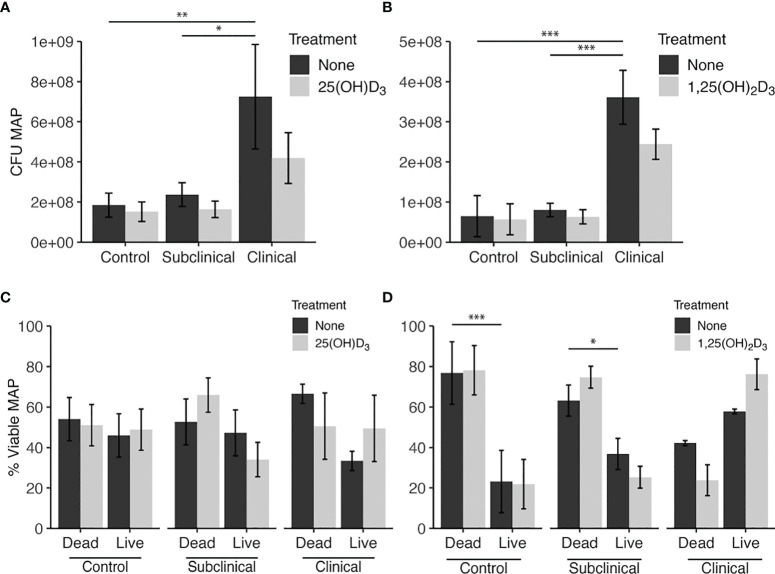
Quantitation of *Mycobacterium avium* subsp. *paratuberculosis* (MAP) present within monocyte-derived macrophages (MDMs) cultured from naturally infected dairy cattle (subclinical n=8, clinical n=8, and noninfected controls n=8). Viable intracellular MAP colony forming units (CFUs) were quantified using PMAxx dye and IS900 gene amplification *via* real time qPCR and standard curve. **(A, B)** show CFU recovered from samples. **(C, D)** show proportion of viable MAP that was calculated from PMAxx viability CFU data divided by the number of MAP inoculated in MDM cultures to achieve a 10:1 MOI. Data are presented as the mean ± SE and significance is defined as *< 0.05, **< 0.01, ***< 0.001.

The PMAxx assay was also used to quantify the amount of live MAP within bovine MDMs using a standard curve generated with serial dilutions of MAP DNA. The results from this assay rely upon a DNA-intercalating, PCR-inhibiting propidium monoazide dye treatment with qPCR output to define the viable population of intracellular MAP. In consensus with the *Bac*Light data, this assay also showed increased (*P* < 0.05) MAP in MDMs from clinical cows in both vitamin D_3_ experiments (**Figures 3A, B**). The proportion of dead MAP in the PMAxx treated samples was extrapolated using the recovered live CFU counts and the total amount of MAP CFU inoculated into each sample to achieve a 10:1 MOI. When expressed as a percentage of viable MAP (**Figures 3C, D**), results suggested a significantly greater proportion of dead MAP in control (*P* < 0.001) and subclinical (*P* < 0.05) cows in the 1,25(OH)_2_D_3_ experiment.

### 3.2 MDM Activation Marker Expression and iNOS Activity

To further understand how macrophage function was impacted by infection status and vitamin D_3_ treatment, markers of cellular activation on MDMs were investigated. MDMs were identified through the positive labeling of CD68, a pan-macrophage marker, and then further analyzed to measure the mean fluorescence intensity (MFI) of CD40 and MHCII expression located on the cell surface.

**Figure 4:**
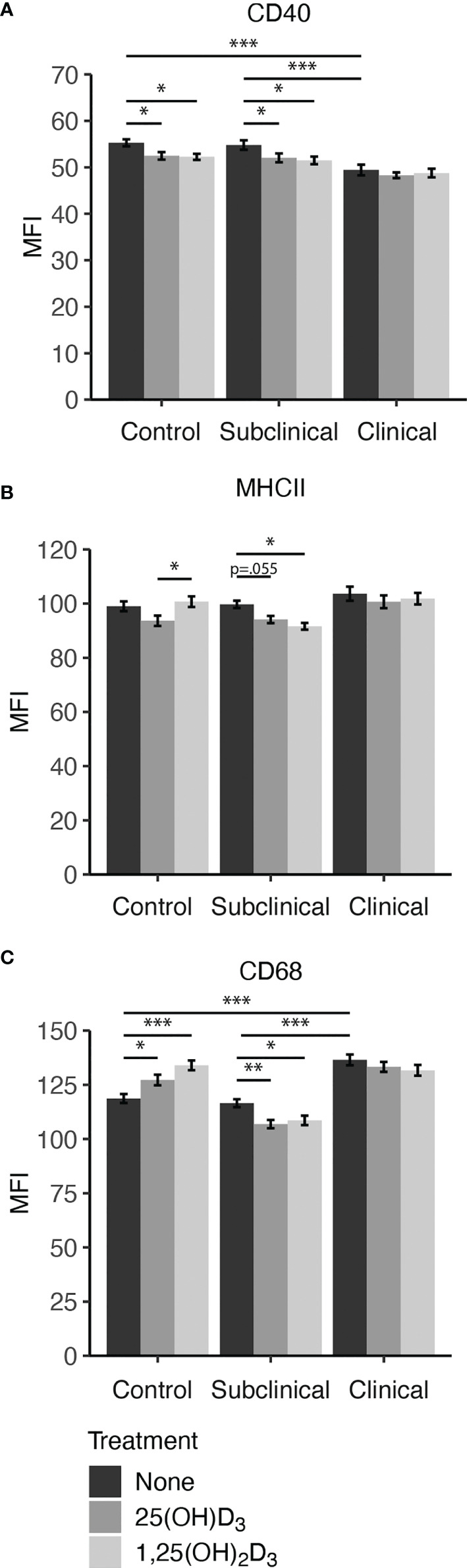
Macrophage expression of activation markers **(A)** CD40, **(B)** MHCII, and pan-macrophage marker **(C)** CD68. Peripheral blood mononuclear cells (PBMCs) were isolated from the whole blood of dairy cattle naturally infected with *Mycobacterium avium* subsp. *paratuberculosis* (MAP) (clinical n=7, subclinical n=8) or noninfected controls (n=6). Cells were cultured 5-6 days to generate monocyte-derived macrophages (MDMs), pre-treated with vitamin D_3_ as detailed in methods, then incubated 24 hrs with live MAP ± 25(OH)D_3_ or ± 1,25(OH)_2_D_3_. The mean fluorescence intensity (MFI) for each target was measured using marker specific primary antibodies coupled with an Alexa Fluor labeled secondary antibody (MHCII[AF488]; CD68[AF594]), or biotin labeled secondary and NeutrAvidin-DyLight 650 tertiary antibody (CD40). Data are presented as the MFI ± SE and significance levels are as follows: *< 0.05, **< 0.01, ***< 0.001.

Our results indicated that infection status influences CD40 expression, showing downregulation in untreated MDMs from clinical cows when compared to controls (*P* < 0.001) and subclinical cows (**Figure 4A**). Both 25(OH)D_3_ and 1,25(OH)_2_D_3_ had a significantly negative effect upon CD40 expression in noninfected controls (*P* < 0.05) and subclinically infected animals (*P* < 0.05). No significant changes in expression were observed in clinical cows following vitamin D_3_ treatment.

Expression of MHCII (**Figure 4B**) was significantly decreased in subclinical cows upon treatment with 1,25(OH)_2_D_3_ (*P* < 0.05) or 25(OH)D_3_ (*P* = 0.055). Control cow MDMs treated with 25(OH)D_3_ had a significant reduction in marker expression compared to 1,25(OH)_2_D_3_ (*P* < 0.05) but was marginally less than untreated MDMs (*P* = 0.071). There was no observed difference between 1,25(OH)_2_D_3_ treatment and untreated control MDMs for this marker within the noninfected control cows. Addition of vitamin D_3_ did not elicit changes in MHCII expression for clinical cows.

**Figure 5:**
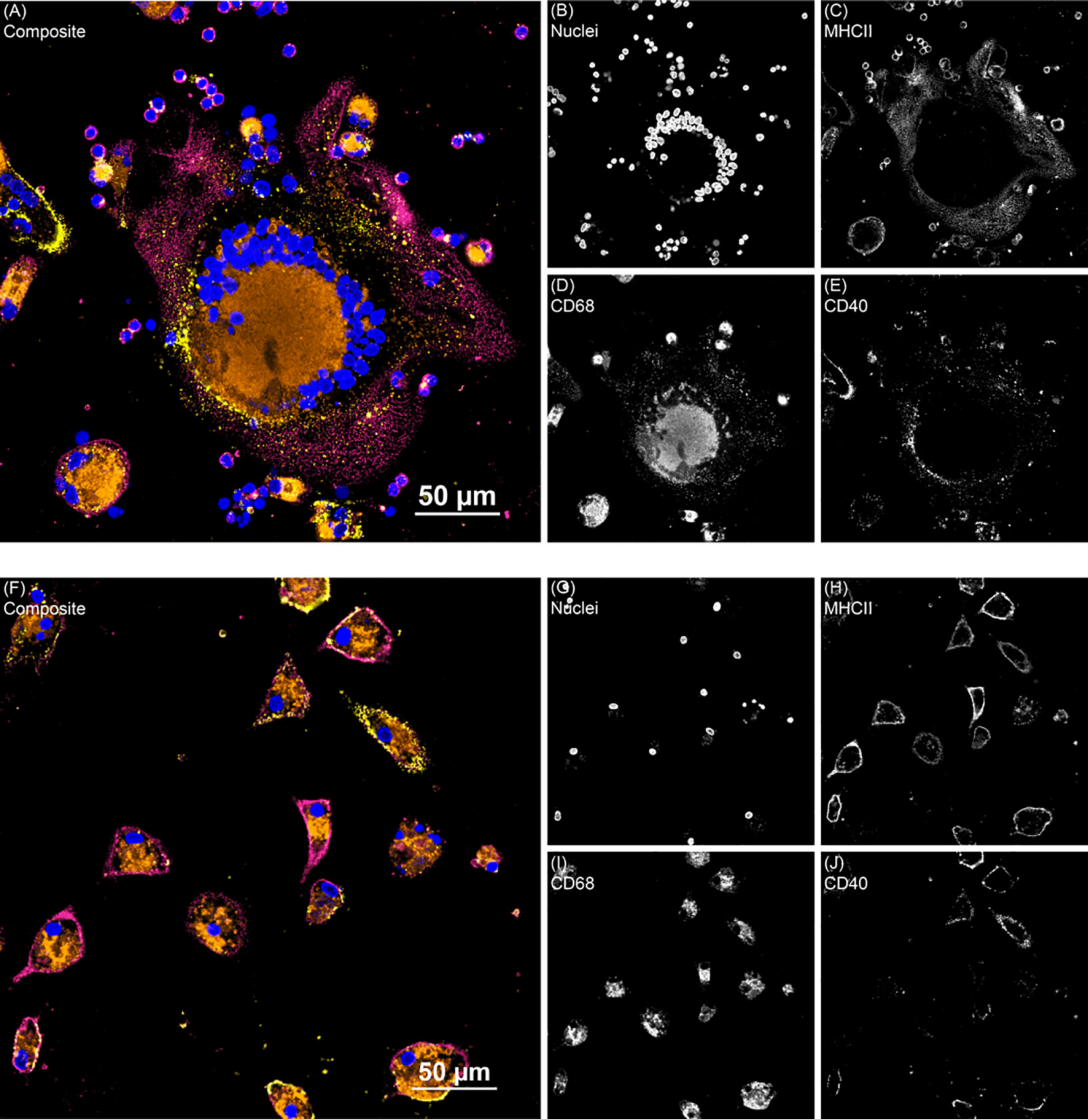
Two representative fluorescence images from the monocyte derived macrophage activation marker experiment. **(A)** Shows giant cell formation from a subclinical cow MDM sample treated with 25(OH)D_3_ and **(F)** shows clinical cow untreated MDMs. Composite images show all channels. **(B, G)** DAPI counterstain was used to detect nuclei (blue) and was excited on the 405 nm laser. **(C, H)** MHCII (magenta) was labeled with an AF488 secondary antibody and excited with the 488 nm laser. **(D, I)** CD68 (orange) was labeled with an AF594 secondary antibody and excited on the 561 nm laser. **(E, J)** CD40 (yellow) was labeled with a NeutrAvidin DyLight 650 tertiary antibody and excited on the 640 nm laser.

Overall, clinical cows had higher (*P* < 0.001) expression of CD68 on untreated MDMs compared to both control and subclinical cows (**Figure 4C**). The inclusion of vitamin D_3_ induced notable changes, with significant increases observed in CD68 expression following treatment with 25(OH)D_3_ (*P* < 0.05) or 1,25(OH)_2_D_3_ (*P* < 0.001) in noninfected control cows. In contrast, cows naturally infected with MAP demonstrated lower CD68 expression upon treatment with vitamin D_3_. Within the MAP infected groups, only subclinical animals had a significant reduction in CD68 expression following treatment with 25(OH)D_3_ (*P* < 0.01) or 1,25(OH)_2_D_3_ (*P* < 0.05). Representative fluorescence images are presented in **Figure 5** that show location of expression for each marker.

**Figure 6:**
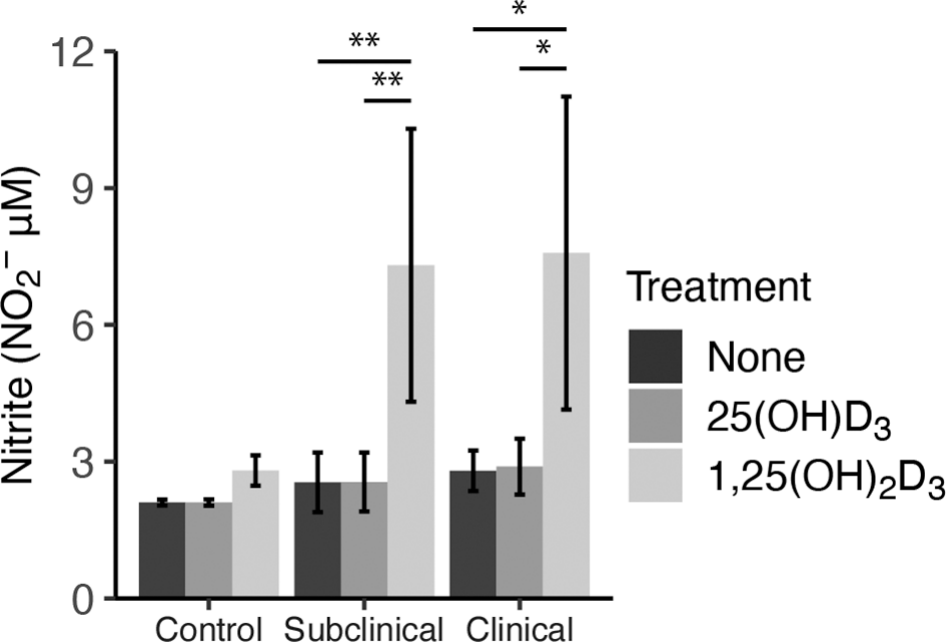
Nitrite production by monocyte-derived macrophages (MDMs) co-cultured with peripheral blood mononuclear cells (PBMCs) from naturally infected dairy cattle (clinical n=7, subclinical n=7) or noninfected controls (n=9). Peripheral blood mononuclear cells (PBMCs) isolated from whole blood were cultured 5-6 days to generate MDMs, pre-treated with vitamin D_3_ presented in methods, then incubated 24 hrs with live MAP (10:1 MOI) ± 25(OH)D_3_ or ± 1,25(OH)_2_D_3_. Nitrite concentrations were determined from cell culture supernatants by competitive binding of sulfanilamide and NED solutions in the Griess assay. Absorbance was measured at 540 nm on a SpectraMax microplate reader. Data are presented as the mean ± SE and significance is defined by *< 0.05, **< 0.01, ***< 0.001.

As an additional measure of MDM activation and function, nitrite production was measured in cell culture supernatants to indirectly assess iNOS activity (**Figure 6**). Cattle infected with MAP were shown to have significantly increased levels of nitrite after treatment with 1,25(OH)_2_D_3_ compared to 25(OH)D_3_ treated and untreated cells (subclinical, *P* < 0.01; clinical, (*P* < 0.05). Control cows followed this same trend, although treatment was not significant. There was no observable difference in any group treated with 25(OH)D_3_.

### 3.3 Cytokine Secretion

**Figure 7:**
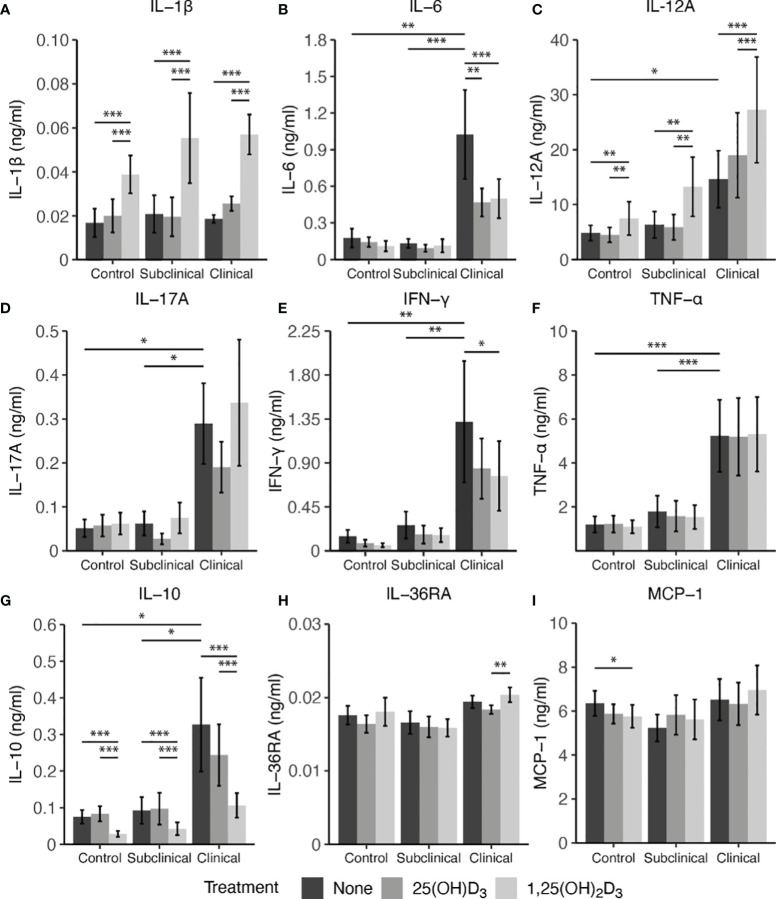
Cytokine expression from monocyte-derived macrophages (MDMs) co-cultured with peripheral blood mononuclear cells (PBMCs) from naturally infected dairy cattle (clinical n=7, subclinical n=7) or noninfected controls (n=9). PBMCs isolated from whole blood were cultured 5-6 days to generate MDMs, pre-treated with vitamin D_3_ as detailed in methods, then incubated 24 hrs with live MAP (10:1 MOI) ± 25(OH)D_3_ or ± 1,25(OH)_2_D_3_. Protein concentrations were determined from cell culture supernatants using Milliplex magnetic bead assay for **(A)** IL-1β, **(B)** IL-6, **(D)** IL-17A, **(E)** IFN-γ, **(F)** TNF-α, **(G)** IL-10, **(H)** IL-36RA, **(I)** MCP-1, while **(C)** IL-12A was measured using standard ELISA. Data are presented as the mean ± SE and significance levels are defined as * < 0.05, ** < 0.01, *** < 0.001.

Secretion of cytokines in MDM culture supernatants was assessed after infection with live MAP for 24 hrs following pre-treatment with 25(OH)D_3_ for 24 hrs or 1,25(OH)_2_D_3_ for 4-6 hrs (**Figure 7**). A significant increase in IL-1β secretion was observed for control (*P* < 0.001), subclinical (*P* < 0.001), and clinical (*P* < 0.001) cows after addition of 1,25(OH)_2_D_3_ when compared to untreated MDMs or those treated with 25(OH)D_3_ (**Figure 7A**). A similar increase in each infection status group was observed for IL-12A secretion following addition of 1,25(OH)_2_D_3_ (**Figure 7C**; *P* < 0.01). Clinical animals produced the greatest amount of IL-12A, being significantly greater (*P* < 0.05) when compared to control cows and trending the same in subclinical cows (*P* = 0.15).

In contrast, treatment of MDMs with 1,25(OH)_2_D_3_ resulted in a significant reduction in IL-10 secretion for both infected cows and noninfected controls (*P* < 0.001) when compared to 25(OH)D_3_ treated or untreated MDMs (**Figure 7G**). There was no difference between untreated and 25(OH)D_3_ treated cells. Levels of IL-10 were also found to be significantly higher (*P* < 0.05) in clinical animals compared to the subclinical and noninfected control groups.

Vitamin D_3_ treatment was also observed to have a significant impact on IL-6 secretion, but only in the clinical group (**Figure 7B**). Clinical cows had significantly higher levels of IL-6 compared to subclinically infected (*P* < 0.001) and healthy control cows (*P* < 0.01). Additionally, MDMs from clinical cows treated with either 25(OH)D_3_ or 1,25(OH)_2_D_3_ resulted in reduced IL-6 secretion (*P* < 0.01 and *P* < 0.001, respectively). IFN-γ production was also highest in clinical cows, being significantly different from both control and subclinical groups (**Figure 7E**; *P* < 0.01). Treatment with 1,25(OH)_2_D_3_ resulted in a significant reduction of IFN-γ production in clinical animals (*P* < 0.05). While no other notable vitamin D_3_ treatment effects were observed for other cytokines investigated (IL-17A, IL-36RA, MCP-1, and TNF-α), infection status did result in measurable differences, with clinical cows secreting higher levels of IL-17A (**Figure 7D**; *P* < 0.05) and TNF-α (**Figure 7F**; *P* < 0.001) compared to the subclinical and noninfected control groups.

### 3.4 Cytokine Gene Expression

**Figure 8:**
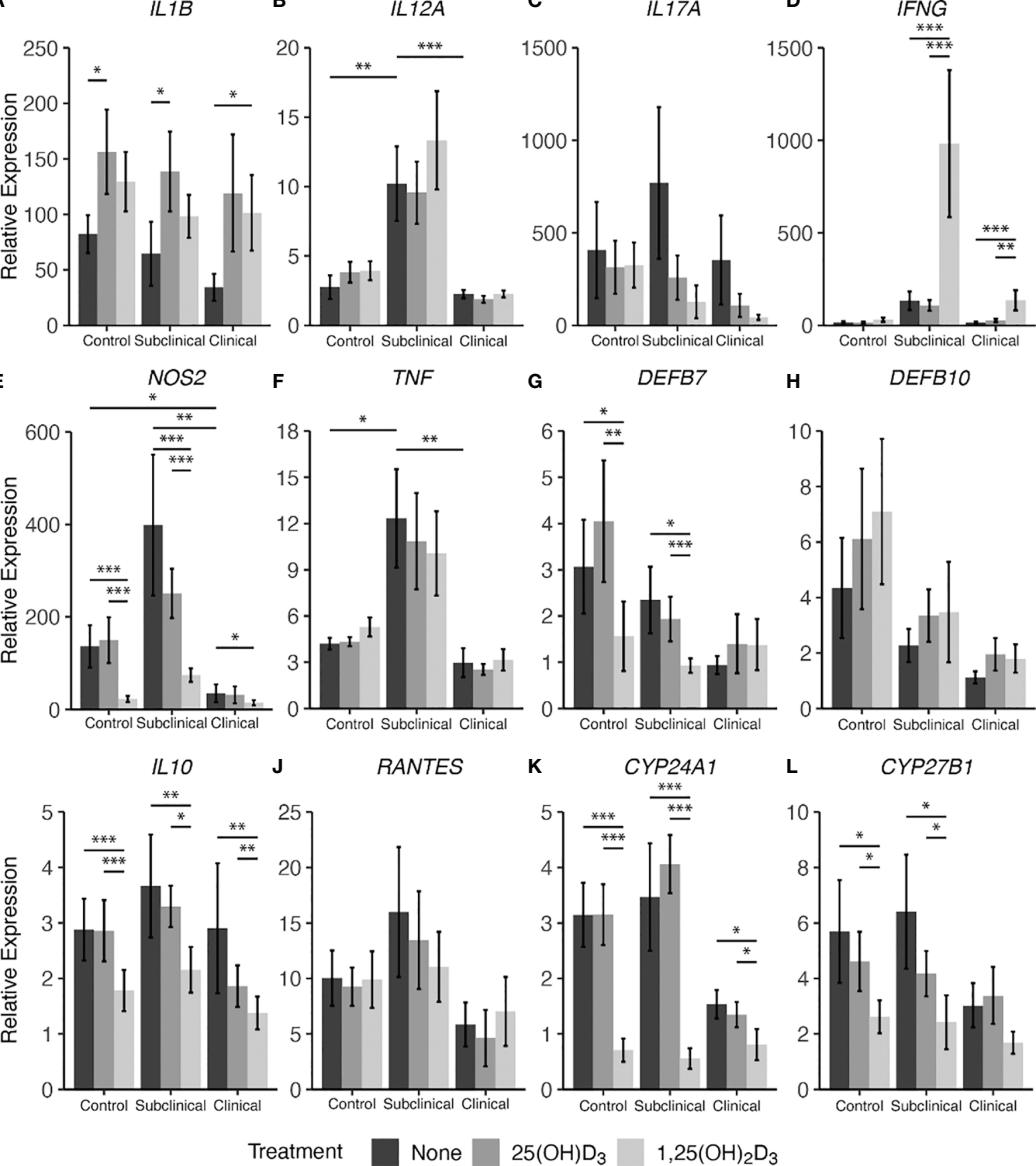
Cytokine gene expression from monocyte-derived macrophages (MDMs) co-cultured with peripheral blood mononuclear cells (PBMCs) from naturally infected dairy cattle (clinical n=7, subclinical n=7) or noninfected controls (n=9). PBMCs isolated from whole blood were cultured 5-6 days to generate MDMs, pre-treated with vitamin D_3_ as detailed in methods, then incubated 24 hrs with live MAP (10:1 MOI) ± 25(OH)D_3_ or ± 1,25(OH)_2_D_3_. Extraction and purification of RNA was performed using Qiagen RNeasy Mini kits and was reverse transcribed with Superscript IV. Gene expression for **(A)** *IL1B*, **(B)** *IL12A*, **(C)** *IL17A*, **(D)** *IFNG*, **(E)** *NOS2*, **(F)** *TNF*, **(G)** *DEFB7*, **(H)** *DEFB10*, **(I)** *IL10*, **(J)** *RANTES*, **(K)** *CYP24A1*, and **(L)** *CYP27B1* were determined using TaqMan assays and was normalized to the eukaryotic 18S rRNA reference gene. Data were transformed using the 2^-ΔΔCt^ method and are presented as the mean relative gene expression (RQ) ± SE compared to each sample’s respective non-stimulated (NS) control. Statistics were performed on ∆∆Ct values and significance levels are as follows: * < 0.05, ** < 0.01, *** < 0.001.

Lastly, cytokine gene expression was investigated complementary to secreted protein levels and the resulting data are shown in **Figure 8**. Notable effects were seen in this experiment on expression of genes that control vitamin D hydroxylases upon vitamin D_3_ treatment of MDMs. Overall, infection status influenced expression of *CYP24A1* (**Figure 8K**), also known as 24-hydroxylase, with an observable decrease in expression for clinical cows compared to controls (*P* = 0.12) and subclinical cows (*P* = 0.06). Additionally, with 1,25(OH)_2_D_3_ treatment a significant reduction in *CYP24A1* expression in MDMs from control (*P* < 0.001), subclinical (*P* < 0.001), and clinical (*P* < 0.05) cows was noted. Similarly, 1α-hydroxylase (*CYP27B1*) gene transcripts were significantly reduced in control (*P* < 0.05) and subclinical (*P* < 0.05) cows with the addition of 1,25(OH)_2_D_3_ (**Figure 8L**). Clinical cows also followed this trend, but the reduction was not significant (*P* = 0.16). 25(OH)D_3_ treatment showed a modest reduction in *CYP27B1* transcripts in control and subclinical cows.

In parallel, 1,25(OH)_2_D_3_ treatment exhibited a downregulatory effect on β-defensin 7 (*DEFB7)* gene expression for control and subclinical cows compared to both untreated MDMs (**Figure 8G**; *P* < 0.05) and MDMs treated with 25(OH)D_3_ (*P* < 0.01 and *P* < 0.001, respectively). In contrast, treatment with vitamin D_3_ appeared to have an upregulatory effect on all infection status groups for β-defensin 10 gene *DEFB10* (**Figure 8H**) and there was a gradual decrease in *DEFB7* and *DEFB10* transcripts with increasing disease severity, with clinical animals expressing the least amount of these gene transcripts; however, no comparisons were significant.

Key cytokine genes that were downregulated by treatment with 1,25(OH)_2_D_3_ were *NOS2* (**Figure 8E**) and *IL10* (**Figure 8I**), demonstrating a significant (*P* < 0.05 to *P* < 0.001) downregulation in all groups of cows. There were no significant differences between 25(OH)D_3_ treated and untreated MDM transcript levels for either of the two aforementioned cytokine genes in any group. Clinical cows were observed to have lower amounts of *NOS2* expression compared to control (*P* < 0.05) and subclinical animals (*P* < 0.01). *IL17A* showed no significant differences among infection status or vitamin D_3_ treatment (**Figure 8C**).

IL-12A (*IL12A*; **Figure 8B**) and TNF-α (*TNF*; **Figure 8F**) genes were expressed at highest levels in subclinical cows, showing significant increases compared to control (*P* < 0.01 and *P* < 0.05) and clinical cows (*P* < 0.001 and *P* < 0.01, respectively). This same trend was also observed in IFN-γ (*IFNG*; Fig 8D) gene expression when compared to control (*P* = 0.06) and clinical cows (*P* = 0.12). The subclinical group also trended higher in *RANTES* (**Figure 8J**) expression compared to clinical animals (*P* = 0.14), but without significance.

Conversely, upregulation of cytokine gene transcripts was also seen for some targets after treatment with 1,25(OH)_2_D_3_. Significantly higher levels of *IFNG* were expressed in both groups of infected animals after treatment of MDMs with 1,25(OH)_2_D_3_ (*P* < 0.01 to *P* < 0.001), while 25(OH)D_3_ had no effect compared to untreated MDMs. Upregulation of IL-1β (*IL1B*; **Figure 8A**) was dependent upon the form of vitamin D_3_, with control and subclinical cows having increased expression of *IL1B* after treatment with 25(OH)D_3_ (*P* < 0.05), and clinically infected animals demonstrating significant upregulation following 1,25(OH)_2_D_3_ treatment (*P* < 0.05). Aggregating data for infected animals resulted in greater *IL1B* expression following 25(OH)D_3_ (*P* < 0.01) or 1,25(OH)_2_D_3_ (*P* = 0.051) treatment when compared to untreated cells.

## 4 Discussion

Animals at subclinical stages of MAP infection retain their ability to maintain Th1 pro-inflammatory cytokine responses that assist with bacterial destruction and clearance (Stabel, 2000; Jenvey et al., 2019). Clinically infected animals tend to be characterized by a predominantly Th2 response and this stage has prominent IL-10 secretion, which has negative regulatory effects upon pro-inflammatory responses (Hussain et al., 2016). These observations are speculated to be related to abrogation of T cell crosstalk with macrophages as a result of a persistently activated state and reduction of antigen presentation (Rathnaiah et al., 2017). Animals at this stage of disease also tend to develop MAP-specific antibodies that do not provide benefits of protection (Khalifeh and Stabel, 2004).

While vitamin D_3_ has its effects solidly established in human infectious disease, particularly *M. tb* (Crowle et al., 1987; Liu et al., 2007), interest in bovine vitamin D status and its effect on disease states has become a topic of interest in recent years. Using a bovine model for Respiratory Syncytial Virus (RSV), the addition of high levels of 25(OH)D_3_ to diets fed to calves increased pro-inflammatory cytokine production in lung lesions, but no lesion resolution outcomes were observed with this treatment (Sacco et al., 2012). Another study has observed protective effects of 25(OH)D_3_ in a dairy cattle mastitis model, with significant reductions in bacterial load and overall clinical symptoms following intramammary experimental challenge with *Streptococcus uberis* and concurrent 25(OH)D_3_ treatment (Lippolis et al., 2011). Little work has been done characterizing links between vitamin D_3_ status and vitamin D_3_ treatment on immune responses from cattle infected with MAP. Cows in the clinical stage of infection have been previously reported to have significantly reduced serum 25(OH)D_3_ levels, possibly due to inhibition of dietary vitamin D_3_ absorption from pathologic intestinal mucosal thickening (Stabel et al., 2019; Wherry et al., 2021).

Effects of vitamin D_3_ treatment on *CYP* gene expression from cows in different stages of paratuberculosis are novel data presented in this study and align similarly with previous work in healthy dairy cattle. Monocytes isolated for stimulation *in vitro* with LPS resulted in a decrease in the 25(OH)D_3_ converting enzyme 1α-hydroxylase (*CYP27B1*) mRNA expression following treatment with 25(OH)D_3_ or 1,25(OH)_2_D_3_ (Nelson et al., 2010b). Downregulation of *CYP27B1* by 1,25(OH)_2_D_3_ was significantly different than LPS stimulation alone or coupled with 25(OH)D_3_ treatment, in agreement with observations made in the present study for treatment with either form of vitamin D_3_ following infection of MDMs with live MAP. These results may indicate that the presence of high levels of 1,25(OH)_2_D_3_ signal to the host cell that sufficient quantities are available for immune system utilization and more does not need to be converted from 25(OH)D_3_. Previous work in bovine tissue showed cows with clinical stage paratuberculosis had greater levels of *CYP27B1* transcripts compared to subclinical cows, which may provide evidence of the immune system attempting to increase the amount of local 1,25(OH)_2_D_3_ available for immunomodulatory action (Stabel et al., 2019). However, the present study presented no significant differences in *CYP27B1* expression between infection status groups. Furthermore, *CYP24A1* is a hydroxylase that functions to regulate 1,25(OH)_2_D_3_ levels through its conversion to a biologically inactive form. The present study observed a significant reduction in expression of this hydroxylase in MDMs infected with live MAP and treated with 1,25(OH)_2_D_3_ for all infection status groups. A previous study utilizing a bovine monocyte model to investigate *CYP24A1* expression following 1,25(OH)_2_D_3_ treatment showed significant upregulation, but following stimulation with LPS this increase in expression was significantly dampened. This may suggest that upon activation of the host cell during infection, 1,25(OH)_2_D_3_ may downregulate CYP24A1 inactivation of 1,25(OH)_2_D_3_ to keep active vitamin D_3_ concentrations high for other regulatory signaling events within the cell. Additionally, the trending decrease in *CYP24A1* expression in clinical cows compared to control and subclinicals could be a compensatory mechanism to maintain adequate 1,25(OH)_2_D_3_ concentrations, and could be related to the previously mentioned reduction in 25(OH)D_3_ serum concentrations reported in clinical cows (Stabel et al., 2019; Wherry et al., 2021).

The present study investigated vitamin D_3_’s role in expression of β-defensins, which are frontline innate host antimicrobial peptides expressed in a wide variety of tissue including epithelial cells and immune cells (Meade et al., 2014). *DEFB7* expression was found to be significantly decreased following 1,25(OH)_2_D_3_ treatment in control and subclinical cows, with clinical cows being unaffected. *DEFB10* expression was not significantly different following treatment with either form of vitamin D_3_; however, a trend of decreasing expression with increasing disease severity was observed and infected animals generally had lower expression than healthy control animals (*P* = 0.168). These observations could indicate that *DEFB10* expression does not contribute to mechanisms of control in MAP infection.

Further analysis of potential antimicrobial agents regulated by vitamin D_3_ showed increased nitrite production upon treatment of MAP infected MDMs with 1,25(OH)_2_D_3_ in the present study. This effect had previously been observed in PBMCs from healthy cattle stimulated with 100 ng/ml LPS (Nelson et al., 2010b), and PBMCs from cattle experimentally infected with *M. bovis* and stimulated *in vitro* with either *M. bovis* cell culture filtrate or *M. bovis* PPD (Waters et al., 2001). A recent study using a bovine monocyte derived macrophage model showed concurrent culture with 1,25(OH)_2_D_3_ and *M. bovis* at an MOI of 2:1 resulted in upregulation of nitric oxide production and *NOS2* expression (García-Barragán et al., 2018). In comparison, the present study observed a similar 1,25(OH)_2_D_3_ dependent increase in nitric oxide production, but a disparate pattern of *NOS2* expression was observed in which there was a significant decrease in all infection status groups following the same 1,25(OH)_2_D_3_ treatment. This might be explained by multiple factors, including co-culture with PBMCs, which would allow opportunities for MDM crosstalk with T cells. Additionally, the nature of the immune cell activating antigen in the present study was a whole, live mycobacterium rather than a soluble antigen, which perhaps employs mRNA expression or translational regulatory mechanisms that have not yet been investigated for MAP. Such mechanisms have been described for *M. tb*, which has been shown to exert alternative splicing events on GTPase Rab8B (*RAB8B*) transcripts in human MDMs resulting in ineffective, truncated transcripts that hinder endosomal trafficking and lysosomal maturation aiding in *M. tb* survival (Kalam et al., 2017).

IL-6 is known as a pleiotropic cytokine depending on the context of disease (Kishimoto, 2006) and its overproduction has been linked to inflammatory disease states. *M. tb* lipoarabinomannan (LAM) has been shown to induce IL-6 and TNF-α production in bovine monocytes (Adams and Czuprynski, 1994) and MAP-infected ileal tissue from cattle has higher IL-6 expression than ileal tissue from noninfected controls (Lee et al., 2001). In the present study, significantly higher IL-6 secretion was observed in clinical animals, but a reduction in IL-6 was noted following treatment with 25(OH)D_3_ or 1,25(OH)_2_D_3_. This is but one example of the diverse compensatory actions that vitamin D_3_ can elicit on the host immune system during infection.

IL-10 modulates pro-inflammatory cytokine responses to allow mycobacterial species to better survive within host cells (Weiss et al., 2005; Redford et al., 2010). The loss of inhibitory mechanisms on inflammation driven by IL-10 from regulatory T cells (Tregs) allows for increased production of pro-inflammatory IL-1β and IL-12 by activated macrophages in the presence of antigenic stimuli (Weiss et al., 2005; Ma et al., 2015; Ipseiz et al., 2020), promoting a Th1-like response. A consistent downregulation of IL-10 gene and protein expression after exposure of MDMs to live MAP and 1,25(OH)_2_D_3_ was observed herein, effects that are contradictory to a previous study showing IL-10 cytokine production increases in human monocyte-derived macrophages infected with *M. tb* following 1,25(OH)_2_D_3_ treatment (Eklund et al., 2013). Interestingly, the downregulation of IL-10 mRNA and protein levels could partially explain the observed increase in dead MAP in clinical cows treated with 1,25(OH)_2_D_3_ from the *Bac*Light viability experiment and may be linked to the upregulation of pro-inflammatory cytokines observed in this study. IL-1β is secreted by monocytes, macrophages, and dendritic cells (Fields et al., 2019) and is recognized as playing a key role in mediating control of *M. tb* infection in humans (Mayer-Barber et al., 2010). It is also expressed at higher levels in tissue from cattle infected with MAP (Lee et al., 2001). Additional work has shown that 1,25(OH)_2_D_3_ treatment of LPS activated bovine monocytes increased *IL1B* mRNA expression (Nelson et al., 2010b), an effect that also aligned with our findings for both mRNA transcripts and protein levels. Complementary findings in 1,25(OH)_2_D_3_ treated human MDMs infected with *M. tb* also show significant upregulation of IL-1β secretion in cell culture supernatants (Eklund et al., 2013). Additional pro-inflammatory responses following 1,25(OH)_2_D_3_ treatment include increased *IFNG* expression in subclinical and clinical cow cell cultures, although trends for IFN-γ protein expression were inconsistent with this pattern. Elevated *IFNG* gene expression in the tissue of clinical cows compared to subclinically infected and noninfected controls has been observed, indicating clinical animals may be in a pro-inflammatory state (Stabel et al., 2019). While the present study showed no significant differences for *IFNG* expression in MDM cultures between infection status groups, patterns of IFN-γ concentrations were similar to the observations made by Stabel et al. (2019) for *IFNG* transcript levels, being significantly greater in clinical animals.

Furthermore, the fluorescence-based viability assay (*Bac*Light) in the present study demonstrated a significant increase in live, dead, and total MAP present within MDMs from clinically infected cows following *in vitro* treatment with exogenous 1,25(OH)_2_D_3_ or 25(OH)D_3_. Previous work has shown the ability of 1,25(OH)_2_D_3_ to modulate immune responses resulting in a reduction of *M. tb* CFU within human MDMs after pre-treatment of cells with 4 μg/ml for 24 hrs, followed by *M. tb* infection accompanied by a second pulse with 1,25(OH)_2_D_3_ (Crowle et al., 1987). This treatment was the highest concentration tested and also conferred the most protection, which became more evident on day 4 when intracellular CFU of *M. tb* began to decrease, which was speculated to be a result of either killing of *M. tb* by the host phagocyte or inhibition of bacilli replication. More recent work has shown that vitamin D can activate antimicrobial activity in both human monocytes and macrophages through the induction of cathelicidin following TLR2/1 heterodimer activation (Liu et al., 2006; Liu et al., 2007). Phagocytic capacity has also been shown to increase in macrophages treated with 1,25(OH)_2_D_3_, perhaps through upregulation of complement receptor immunoglobulin (CRIg) (Small et al., 2021). This mechanism could support the finding in our study showing a significant increase in total MAP uptake for clinical animals, as other recent reports have described CRIg functioning as a pattern recognition receptor (PRR) for gram-negative bacteria and parasites (Zeng et al., 2016; Liu et al., 2019).

Granulomatous tissue lesions in cattle infected with *M. bovis* have been shown to have a greater number of CD68+ cells associated with inflammation (Wangoo et al., 2005), as well as ileal tissue from cattle naturally infected with MAP (Lee et al., 2001). In the present study, clinical animals showed a positive correlation between the total number of MAP phagocytized and CD68 expressed on untreated MDMs. This observation may be partially explained by previous reports that have shown CD68 expression is mainly localized to lysosomal and endosomal compartments within the macrophage (Holness and Simmons, 1993). While vitamin D_3_ treatments did not result in a correlative change between CD68 expression and phagocytosis of MAP, it is likely other cell surface receptors not investigated in this study assist in modulating this process. The current study further investigated markers of activation, represented by CD40 and MHCII, expressed on CD68+ MDMs. The CD40/CD40L signaling pathway is important in the modulation of Th1 cytokine responses (McDyer et al., 1998). The significant decrease in CD40 expression on MDMs from clinical cows is interesting and may indicate that as MAP infection progresses, macrophages play a lesser role in the induction of some key T cell mediated pro-inflammatory responses. Previous work in MAP infected cattle showed high levels of CD40 expression on CD4+ T cells, with expression also being seen on B cells, CD8+ T cells, and γδ T cells (Khalifeh and Stabel, 2013). Work in a murine model show the capacity of CD40 to serve as a co-stimulatory molecule in T cell activation and cytokine signaling (Munroe and Bishop, 2007). Other studies investigating dendritic cells in humans have shown 1,25(OH)_2_D_3_ decreases expression of CD40 and MHCII at a concentration of about 4 ng/ml (Wahono et al., 2014), consistent with the observations in this study although downregulation of MHCII was significant only in the subclinical group while expression in the clinical group trended downward. Other work in humans and cattle also showed 1,25(OH)_2_D_3_ downregulation of MHCII expression in monocytes and monocyte-derived dendritic cells, respectively (Xu et al., 1993; Corripio-Miyar et al., 2017).

Taken together, the data herein show a complex pro- and anti-inflammatory dynamic for vitamin D_3_ treatment of monocyte derived macrophages in the presence of PBMCs. As previously mentioned, prior work done by our group has shown clinical animals to possess significantly lower levels of circulating 25(OH)D_3_ (Stabel et al., 2019; Wherry et al., 2021) and while a critical threshold has not been defined for vitamin D_3_ deficiency and insufficiency, it is plausible for this observation to be correlated with the general pro-inflammatory cytokine profile observed in this infection status group. In the current study, 1,25(OH)_2_D_3_ appeared to be the main form of vitamin D_3_ that modulated significant cytokine responses and nitrite production, and its treatment appeared to elicit similar responses among all infection status groups within each marker. However, clinical animals were shown to increase their phagocytic capacity, resulting in a significantly greater proportion of dead MAP bacilli. Macrophage activation markers CD40 and MHCII were not significantly downregulated in clinical cows, contrary to control and subclinical cows, perhaps contributing to this observation in decreased intracellular MAP viability. Complementary to these results, clinically infected cows overall had a more robust pro-inflammatory cytokine profile, as previously mentioned, exhibiting significantly higher secretion of IL-6, IL-12A, IL-17A, IFN-γ, and TNF-α. Upregulation of some pro-inflammatory cytokines (IL-1β, IL-12A) and increases in iNOS activity through higher levels of nitrite following 1,25(OH)_2_D_3_ may also partially explain the increased capacity of macrophages from clinical cows to control intracellular MAP infection.

Further investigation of the multifaceted host-pathogen interactions during different stages of MAP infection and the potential role of vitamin D_3_ in controlling dissemination of MAP could lead to a better understanding of the timing of events that allow for progression of disease. More studies are needed to identify specific key events within the complex interplay of cytokine signaling that hinder host clearance of MAP and what role each form of vitamin D_3_ plays in mitigation of these host responses.

## Data Availability Statement

The original contributions presented in the study are included in the article/supplementary material. Further inquiries can be directed to the corresponding author.

## Ethics Statement

The animal study was reviewed and approved by NADC Animal Care and Use Committee.

## Author Contributions

Experimental design was conceived by JS, TW, and RD. Experiments were performed by TW. Data analysis was performed by TW and EC. First draft manuscript was prepared by TW. JS, TW, RD, EC, JB, and SM contributed to manuscript revisions. All authors contributed to the article and approved the submitted version.

## Funding

This study was funded through USDA-ARS, CRIS Project 5030-32000-221.

## Conflict of Interest

The authors declare that the research was conducted in the absence of any commercial or financial relationships that could be construed as a potential conflict of interest.

## Publisher’s Note

All claims expressed in this article are solely those of the authors and do not necessarily represent those of their affiliated organizations, or those of the publisher, the editors and the reviewers. Any product that may be evaluated in this article, or claim that may be made by its manufacturer, is not guaranteed or endorsed by the publisher.

## Footnotes

1 Wherry, T. L. T., Mooyottu, S., and Stabel, J. R. (2021). Effects of 1,25-dihydroxyvitamin D3 and 25-hydroxyvitamin D3 on PBMCs From Dairy Cattle Naturally Infected With Mycobacterium avium ssp. Paratuberculosis. Submitted for publication.
