# Corrigendum: Exogenous Vitamin D_3_ Modulates Response of Bovine Macrophages to *Mycobacterium avium* subsp. *paratuberculosis* Infection and Is Dependent Upon Stage of Johne’s Disease

**DOI:** 10.3389/fcimb.2022.876622

**Published:** 2022-03-09

**Authors:** Taylor L.T. Wherry, Rohana P. Dassanayake, Eduardo Casas, Shankumar Mooyottu, John P. Bannantine, Judith R. Stabel

**Affiliations:** ^1^Infectious Bacterial Diseases, National Animal Disease Center, United States Department of Agriculture-Agricultural Research Service (USDA-ARS), Ames, IA, United States; ^2^Department of Veterinary Pathology, Iowa State University, Ames, IA, United States; ^3^Ruminant Diseases and Immunology, National Animal Disease Center, United States Department of Agriculture-Agricultural Research Service (USDA-ARS), Ames, IA, United States

**Keywords:** *Mycobacterium avium* subsp. *paratuberculosis*, cattle, vitamin D, macrophage, immune responses, Johne’s disease

**Incorrect Author Name**

An author name was incorrectly spelled as **Rohana Dassanayake**. The correct spelling is **Rohana P. Dassanayake**.

The authors apologize for this error and state that this does not change the scientific conclusions of the article in any way. The original article has been updated.

## Publisher’s Note

All claims expressed in this article are solely those of the authors and do not necessarily represent those of their affiliated organizations, or those of the publisher, the editors and the reviewers. Any product that may be evaluated in this article, or claim that may be made by its manufacturer, is not guaranteed or endorsed by the publisher.

